# Identifying Metabolomic Profiles of Insulinemic Dietary Patterns

**DOI:** 10.3390/metabo9060120

**Published:** 2019-06-24

**Authors:** Fred K. Tabung, Raji Balasubramanian, Liming Liang, Steven K. Clinton, Elizabeth M. Cespedes Feliciano, JoAnn E. Manson, Linda Van Horn, Jean Wactawski-Wende, Clary B. Clish, Edward L. Giovannucci, Kathryn M. Rexrode

**Affiliations:** 1 Division of Medical Oncology, Department of Internal Medicine, The Ohio State University College of Medicine, Columbus, OH 43210, USA; Steven.Clinton@osumc.edu; 2 The Ohio State University Comprehensive Cancer Center—Arthur G. James Cancer Hospital and Richard J. Solove Research Institute, Columbus, OH 43210, USA; 3 Department of Nutrition, Harvard T.H. Chan School of Public Health, Boston, MA 02115, USA; egiovann@hsph.harvard.edu; 4 Division of Women’s Health, Brigham and Women’s Hospital, Boston, MA 02115, USA; krexrode@bwh.harvard.edu; 5 Department of Biostatistics and Epidemiology, University of Massachusetts-Amherst, MA 01003, USA; rbalasub@umass.edu; 6 Department of Epidemiology, Harvard T.H. Chan School of Public Health, Boston, MA 02115, USA; lliang@hsph.harvard.edu (L.L.); jmanson@rics.bwh.harvard.edu (J.E.M.); 7 Department of Biostatistics, Harvard T.H. Chan School of Public Health, Boston, MA 02115, USA; 8 Division of Research, Kaiser Permanente Northern California, Oakland, CA 94612, USA; Elizabeth.M.Cespedes@kp.org; 9 Division of Preventive Medicine, Brigham and Women’s Hospital, Boston, MA 02115, USA; 10 Harvard Medical School, Boston, MA 02115, USA; 11 Department of Preventive Medicine, Northwestern University, Chicago, IL 60611, USA; lvanhorn@northwestern.edu; 12 Department of Epidemiology and Environmental Health, University at Buffalo, The State University of New York, Buffalo, NY 14214, USA; jww@buffalo.edu; 13 Broad Institute of the Massachusetts Institute of Technology and Harvard University, Cambridge, MA 02142, USA; clary@broadinstitute.org

**Keywords:** dietary patterns, insulinemic diets, metabolomics, postmenopausal women

## Abstract

The food-based empirical dietary index for hyperinsulinemia (EDIH) score assesses the insulinemic potential of diet. This cross-sectional study evaluated associations between EDIH scores from food frequency questionnaires with c-peptide concentrations and with 448 metabolites, from fasting plasma samples, in multivariable linear regression analyses. Metabolites were measured with liquid chromatography tandem mass spectroscopy. Using a robust two-stage study design, discovery of metabolite associations was conducted among 1109 Women’s Health Initiative (WHI) Hormone Therapy (HT) trial participants and results replicated in an independent dataset of 810 WHI Observational Study (OS) participants. In both discovery and replication datasets, statistical significance was based on the false-discovery rate adjusted *P* < 0.05. In the multivariable-adjusted analyses, EDIH was significantly associated with c-peptide concentrations among 919 women (HT & OS) with c-peptide data. On average, c-peptide concentrations were 18% higher (95% CI, 6%, 32%; *P*-trend < 0.0001) in EDIH quintile 5 compared to quintile 1. Twenty-six metabolites were significantly associated with EDIH in the discovery dataset, and 19 of these were replicated in the validation dataset. Nine metabolites were found to decrease in abundance with increasing EDIH scores and included: C14:0 CE, C16:1 CE, C18:1 CE, C18:3 CE, C20:3 CE, C20:5 CE, C36:1 PS plasmalogen, trigonelline, and eicosapentanoate, whereas the 10 metabolites observed to increase with increasing EDIH scores were: C18:2 SM, C36:3 DAG, C36:4 DAG-A, C51:3 TAG, C52:3 TAG, C52:4, TAG, C54:3 TAG, C54:4 TAG, C54:6 TAG, and C10:2 carnitine. Cholesteryl esters, phospholipids, acylglycerols, and acylcarnitines may constitute circulating metabolites that are associated with insulinemic dietary patterns.

## 1. Introduction

Diet has been shown to influence chronic disease development and progression, but the specific biological mechanisms through which diet influences disease risk are not fully understood. However, excess insulin secretion may be one such pathway. Thus, dietary patterns associated with sustained high insulin secretion and blood concentrations may also predispose to higher risk of certain chronic diseases and morbidity and mortality. We have developed an empirical dietary index for hyperinsulinemia (EDIH) score [1,2], in a sample of the Nurses’ Health Study (NHS), and evaluated its construct validity in two independent cohorts of health professionals [1,2]. Our goal was to empirically create a score to assess the insulinemic potential of whole diets defined using food groups. Higher EDIH scores, indicating dietary patterns that tend to lead to higher insulin secretion, have been associated with higher risk of developing colorectal cancer [3] and other cancers of the gastrointestinal track [4]. While it is important to know that the insulinemic potential of the diet is implicated in chronic disease development, identifying specific biological pathways through which diet and insulin may act to influence disease development will further elucidate mechanisms of action to inform effective prevention and intervention strategies. Integrating diet and metabolomics data may be helpful in this regard.

Metabolomic approaches, compared to single analytes, allow for a broader view of multiple pathways implicated in the pathophysiology of disease, by profiling multiple metabolites in biofluids, cells, and tissues. Metabolomics therefore has the potential to identify new biomarkers and provide insights on the biological pathways underlying disease development and progression. In previous population-based prospective studies, global metabolomics profiling has been used to predict the incidence and progression of several diseases including cardiovascular disease [5,6,7], cancer [8,9,10], type 2 diabetes [11,12] and all-cause mortality [13]. Some of these population-based studies have aimed to identify new or novel biomarkers of disease or metabolite profiles that may be influenced and modulated by different human exposures such as diet and lifestyle [14,15]. 

It is well established that insulin secretion and resistance are influenced by adiposity, and high insulin secretion may be a central initiator of insulin-related weight gain [16]. Before the development of clinically significant metabolic diseases, individuals often show dyslipidemia, elevated fasting insulin and insulin resistance [17]. Insulin is the principal anabolic hormone responsible for tissue uptake and storage of energy rich nutrients following ingestion of a meal, and previous studies have documented a positive relationship between obesity status and elevated insulin concentrations in animal models and humans [18]. In this cross-sectional study in a population of postmenopausal women, our objectives were 3-fold: 1) To evaluate the construct validity of the EDIH score in the Women’s Health Initiative (WHI), 2) to use the score to characterize the metabolomic profiles of insulinemic dietary patterns, and 3) to examine the role of body weight.

## 2. Results

### 2.1. EDIH Validation Study

Among the 919 women with c-peptide data, included in the EDIH construct validation study (Table 1), women with dietary patterns with the lowest insulinemic potential (EDIH quintile 1) had lower c-peptide concentrations, lower BMI and reported higher physical activity levels, compared to those consuming hyperinsulinemic diets (EDIH quintile 5). The proportions of overweight or obese women, African Americans, and those with lower educational levels increased across EDIH quintiles, (Table 1). The Spearman correlation coefficient between EDIH scores and c-peptide concentrations was 0.26, *p* < 0.0001, and the EDIH multivariable model explained 16% (r-square) of the variance in c-peptide. 

Table 2 shows the absolute and relative concentrations of plasma c-peptide in quintiles of the EDIH score. In multivariable-adjusted models, the relative difference in plasma c-peptide concentrations was 26% (95% CI, 12%, 42%; *P*-trend < 0.0001) higher in EDIH quintile 5 compared to quintile 1. When additionally adjusted for BMI, the association was attenuated but remained statistically significant: 18% (95% CI, 6%, 32%; *P*-trend < 0.0001). When models were stratified by BMI and additionally adjusted for continuous BMI within BMI strata (*P*-interaction = 0.02), the association was no longer significant among normal weight women (BMI: 15 to <25 kg/m^2^, n = 340; 8%; 95% CI, -11%, 30%; *P*-trend = 0.09); whereas among overweight or obese women (BMI: 25 to 50 kg/m^2^, *n* = 579), plasma c-peptide concentration was 23% (95% CI, 6%, 42%; *P*-trend = 0.0005) higher in EDIH quintile 5 compared to quintile 1 (Table 2).

### 2.2. Metabolomic Profiles of Insulinemic Diets

#### 2.2.1. Characteristics of the EDIH Metabolomics Study Population 

Table S1 (in supplementary materials) presents characteristics of the 1919 women in the metabolomics study, by EDIH quintiles, and the characteristics are quite similar to those in the validation study (*n* = 919), though a separate sample. In both the discovery (*n* = 1109) and replication (*n* = 810) datasets, women consuming diets with the lowest insulinemic potential (EDIH quintile 1) had lower BMI, lower glucose concentrations, lower triglycerides, higher physical activity levels and higher high-density lipoprotein cholesterol concentrations compared to those consuming hyperinsulinemic diets (quintile 5). The proportions of overweight or obese women, African Americans, Hispanic/Latinos, and those with lower educational levels increased across EDIH quintiles. We note that both the validation and metabolomics study populations were similar to the overall WHI population in terms of several demographic and lifestyle factors including education, race (though ancillary biomarker studies tended to oversample minority populations), and BMI. For example, the proportion of African Americans was 10.8% in the WHI-dietary modification trial, 8.2% in the WHI-OS [19,20], 8.7% in the current EDIH validation subsample (comprised of both WHI-CT and WHI-OS), 9.9% in the discovery subsample (WHI-CT) and 12.7% in the metabolite validation subsample (WHI-OS).

In terms of weekly food intake, women with low insulinemic diets consumed on average three servings less processed meat, 11 servings less sugar-sweetened beverages, three servings less fries and 1.5 servings less red meat than women classified with hyperinsulinemic diets. In addition, women with low insulinemic diets consumed on average 13 glasses more wine, 1.6 cups more tea or coffee, three servings more fruit and two servings more green leafy vegetables than the women with hyperinsulinemic dietary patterns. Servings are defined in Table 3, footnote #3. 

#### 2.2.2. Two-stage Discovery and Replication of Metabolites Associated with Insulinemic Diets

In the discovery dataset, the EDIH score was associated with 126 metabolites at an FDR adjusted *P*-value < 0.05 in multivariable-adjusted models without BMI, and after additionally adjusting for BMI, 26 metabolites remained significantly associated (Table 4). Of the 26 metabolites, 19 were replicated in the WHI-OS after adjustment for all covariates (including BMI). The nine metabolites with inverse associations with the EDIH score included: C14:0 CE, C16:1 CE, C18:1 CE, C18:3 CE, C20:3 CE, C20:5 CE, C36:1 PS plasmalogen, trigonelline, and eicosapentanoate. In contrast, the 10 metabolites that were positively associated with EDIH, included the following: C18:2 SM, C36:3 DAG, C36:4 DAG-A, C51:3 TAG, C52:3 TAG, C52:4, TAG, C54:3 TAG, C54:4 TAG, C54:6 TAG, and C10:2 carnitine (Table 4). 

**Figure 1:**
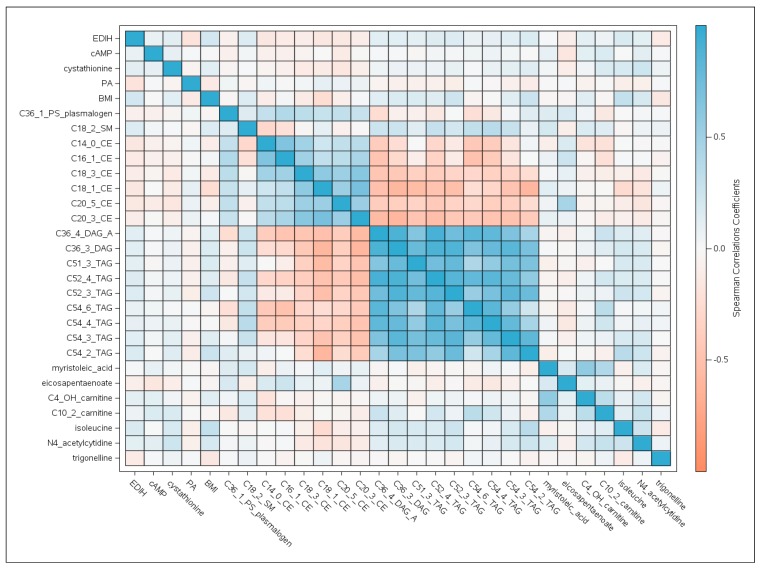
Heat map in the replication dataset showing Spearman correlations between the 26 discovered metabolites and EDIH score, BMI and physical activity (PA). Ordering is by hierarchical clustering. BMI, body mass index; cAMP, cyclic adenosine monophosphate; CE, cholesterol ester; DAG, diacylglycerol; EDIH, empirical dietary index for hyperinsulinemia score; PA, physical activity; TAG, triacylglycerol.

Figure 1 is a heat map in the replication dataset showing Spearman correlations (*r*) between the 26 metabolites and EDIH score, BMI and physical activity; ordered by hierarchical clustering. The cholesterol esters were highly clustered and showed inverse associations with EDIH and BMI and positive associations with physical activity. The strongest inverse correlations with the EDIH were shown by physical activity, −0.16; C14:0 CE, –0.14; and C16:1 CE, –0.12 (all *P* < 0.0001). The diglycerides and triglycerides were also highly clustered and positively associated with EDIH and BMI, and inversely associated with physical activity. The strongest positive correlations with the EDIH were shown by BMI, 0.20; C52:3 TAG, 0.14; and C52:4 TAG, 0.14 (all *P* < 0.0001) (Figure 1). 

#### 2.2.3. Among Underweight and Normal Weight Women (BMI: 15 to <25 kg/m^2^, *n* = 630)

Among underweight (*n* = 20) and normal weight (*n* = 610) women, after adjustment for covariates, including continuous BMI; 12 metabolites were associated with the EDIH score at FDR adjusted *P* < 0.05 and an additional 17 metabolites at an FDR adjusted *P* < 0.10. The 12 metabolites that were significant at an FDR adjusted *P* < 0.05 included seven of the 19 metabolites replicated in the main analyses, namely C16:1 CE, C18:2 SM, C14:0 CE, C54:4 TAG, C36:4 DAG-A, C36:1 PS plasmalogen, C54:6 TAG; and an additional three replicated metabolites from the main analyses (C20:5 CE, C51:3 TAG and C54:3 TAG) were significant at an FDR adjusted *P* < 0.10 (Table 5). In an exploratory analysis among normal weight women in the discovery dataset (*n* = 317), 37 metabolites were associated with EDIH at the nominal *P* < 0.05. Of these, only two (C18:2 SM, C16:1 CE) met the threshold of an FDR-adjusted *P* < 0.05 and an additional two (C32:1 PC, C14:0 CE) satisfied an FDR-adjusted *P* < 0.20 (Table S2); due to limitations of statistical power, no independent replication was conducted among normal weight women.

#### 2.2.4. Among Overweight and Obese Women (BMI: 25 to 50 kg/m^2^, *n* = 1289)

Among overweight or obese women, after adjustment for covariates, including continuous BMI, 64 metabolites were significantly associated with the EDIH score (FDR adjusted *P* < 0.05). All 19 replicated metabolites were among the 64 and were among the top nine hits (Table 6). Of the 64 metabolites, 38 were inversely associated with EDIH and included cholesterol esters, phosphatidylcholines, lysophosphatidylcholines, lysophosphatidyletanolamines and sphingomyelins; whereas the 26 positively associated metabolites were mostly glycerides and carnitines. In an exploratory analysis among overweight and obese women restricted to the metabolite discovery dataset (*n* = 792), we found 83 metabolites to be significantly associated with EDIH at an FDR-adjusted *P* < 0.20 (Table S3). In the replication dataset (Observational Study: *n* = 497), 23 of these metabolites remained associated with the EDIH score at an FDR-adjusted *P* < 0.05 and an additional 10 metabolites at an FDR-adjusted *P* < 0.10. Fourteen of the 19 metabolites replicated in the main analyses were also discovered and replicated in these exploratory analyses at an FDR-adjusted *P* < 0.05, and an additional four metabolites at an FDR-adjusted *P* < 0.20 (Table S3). 

**Figure 2:**
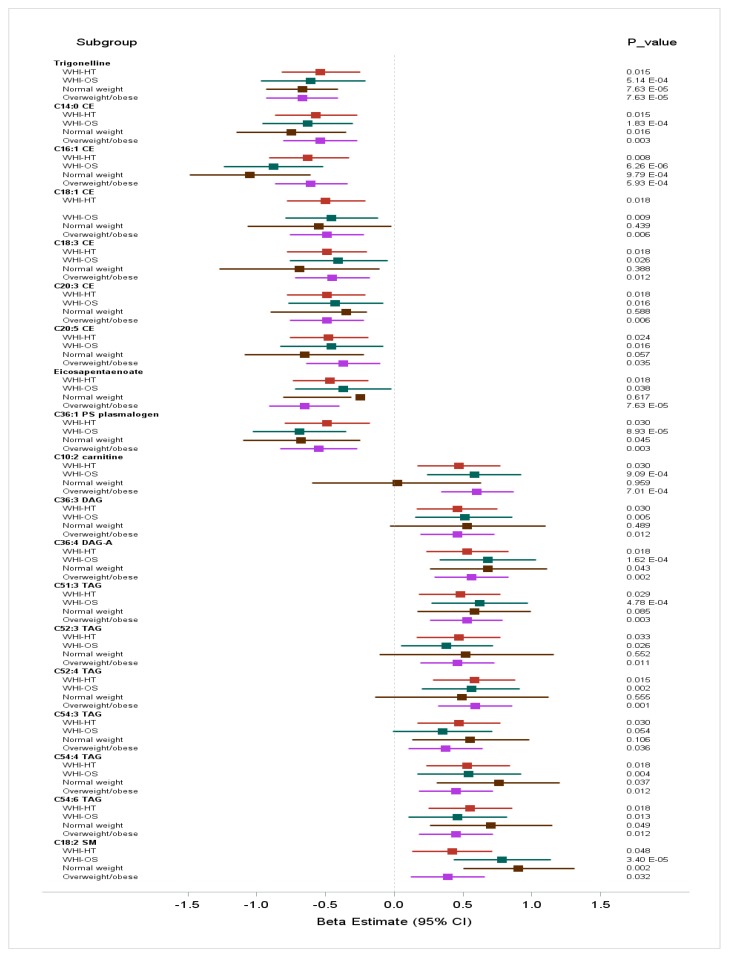
Summary of EDIH associations with the 19 replicated metabolites in the discovery (WHI-HT) and replication (WHI-OS) datasets and in body mass index categories. Associations were adjusted for body mass index (continuous) age, physical activity, educational level, race/ethnicity, aspirin/NSAIDs use, smoking status, WHI Hormone Therapy trial arm, and CHD case-control status. *P*-value is the FDR-adjusted *p*-value.

A summary of the associations of the 19 replicated metabolites in the discovery and replication datasets and in the BMI categories, is presented in Figure 2. 

## 3. Discussion

In the current study, we showed that higher scores of the empirical dietary index for hyperinsulinemia (EDIH) were significantly associated with higher c-peptide concentrations in the WHI; further demonstrating its validity for use in cohorts different than the one in which the score was initially developed. Associations were stronger among overweight or obese women than among normal weight women, in line with findings from previous studies of EDIH and c-peptide concentrations [1,2]. In addition, using a robust 2-stage methodology, we identified and replicated 19 metabolites associated with the insulinemic potential of diet, after adjusting for total energy intake, race/ethnicity, BMI, physical activity, smoking and other potential confounding variables. The 19 metabolites were comprised mainly of acylcarnitines and acylglycerols (diacylglycerols-DAG and triacylglycerols-TAG) that were associated with higher dietary insulinemic potential, and cholesterol esters and plasmalogens, associated with lower dietary insulinemic potential. In line with the differences by BMI observed in the association of EDIH and c-peptide concentrations in the validation study, body weight appeared to modify EDIH associations with metabolite abundances. For example, among normal weight women, 12 metabolites were associated with the EDIH score at an FDR adjusted *P* < 0.05, including seven of the 19 metabolites replicated in the primary analyses; whereas among overweight or obese women, up to 64 metabolites were significantly associated with the EDIH score at an FDR adjusted *P* < 0.05, including all 19 replicated metabolites. Though we observed five times more significant associations among overweight or obese women than among normal weight women, it is possible that some of the differences may be attributed to sample size that was twice higher among overweight or obese women. We note however, that we observed more significant associations in the overweight/obese sample than among the overall study population, including in the exploratory analyses with much smaller samples.

The low insulinemic dietary pattern is high in whole fruit, wholegrains, high-fat dairy products (e.g., cheese, whole milk, ice cream), green-leafy vegetables, coffee, wine, and low in red meat, processed meat, sugar-sweetened beverages, poultry; and has been shown in the current study and elsewhere [2,16] to have a nutrient profile high in fiber, calcium, total carbohydrates, and low in cholesterol, total protein and total fat (including saturated fat-SFA). Therefore, while not strictly a vegetarian diet, the low insulinemic dietary pattern is characteristic of a plant-based dietary pattern. In the current study, the nine replicated metabolites that were higher with a low insulinemic dietary pattern included one omega-3 PUFA (eicosapentaenoate-EPA), one phospholipid plasmalogen (C36:1 PS plasmalogen), one coffee-related alkaloid (trigonelline) and up to six cholesterol esters that included cholesteryl oleate (C18:1 CE) and cholesteryl palmitoleate (C16:1 CE). The cholesterol esters may be indicative of the plant-based nature of low EDIH diets [21]. A study that investigated whether vegan diet improves the metabolic pathway of TAG-rich lipoproteins, consisting in lipoprotein lipolysis and removal of the resulting remnants from circulation, found that remnant removal, estimated by cholesteryl oleate clearance, was significantly faster in vegans compared to omnivores [22]. Vegans showed better regulation of the metabolism of TAG-rich lipoproteins, because they were more efficient in removing remnants that are potentially atherogenic [23]. Low EDIH diets may also reduce the residence time of chylomicron remnants [24], which may have favorable effects in relation to chronic disease development. A lipidomics and transcriptomics analysis in paired prostate cancer tumor and adjacent non-tumor tissues revealed cholesteryl oleate with the highest ability in distinguishing prostate cancer from non-malignant tissue or benign prostatic hyperplasia [25]. Cholesteryl palmitoleate derives from palmitoleic acid and higher concentrations of palmitoleic acid in blood or adipose tissue are consistently associated with a many undesirable outcomes including obesity and metabolic syndrome [26], hypertriglyceridemia [27], type 2 diabetes [28], prostate cancer [29], among other diseases. It is therefore possible that a habitual low insulinemic dietary pattern may reduce risk of certain chronic diseases through its greater efficiency in clearing the blood stream of the remnants of lipid metabolism. 

A low EDIH diet was also favorably associated with EPA. Studies have shown that diets with greater intake of EPA and docosahexaenoic (DHA) fatty acids may promote positive effects, especially on TAG levels and increase high-density lipoprotein (HDL) concentrations [30] which may decrease lipogenic activity and release adiponectin; which is associated with weight loss. Indeed, one of our previous studies showed that long-term dietary changes towards a low insulinemic dietary pattern were associated with less weight gain compared to no or minimal dietary changes [16]. A low EDIH diet may diminish TAG levels via reduced hepatic production of very low-density lipoprotein (VLDL) cholesterol and by increasing lipoprotein lipase activity in chylomicron clearance [31]. Trigonelline, an alkaloid in coffee, also associated favorably with the low EDIH diet. Trigonelline is degraded to an extent during coffee roasting to produce vitamin B3 (niacin), which may suppress colonic inflammation [32] and colon carcinogenesis in mice [33]. In addition, a randomized crossover trial that enrolled 15 overweight men to evaluate the acute effects of coffee and major coffee compounds including trigonelline and chlorogenic acid, found that these two metabolites reduced early glucose and insulin secretion [34].

In contrast, the foods contributing to a hyperinsulinemic dietary pattern include red meat, processed meat, cream soups, margarine, butter, French fries, sugar-sweetened beverages, poultry and eggs [1], and the resulting nutrient profile is rich in total and saturated fat, cholesterol and total protein, and low in fiber and total carbohydrates. The ten replicated metabolites that were associated with a high insulinemic dietary pattern in the current study included one sphingolipid (C18:2 SM), one acylcarnitine (C10:2 carnitine), two DAGs, and five TAGs. The acylglycerols may be generally indicative of the meat-based components contributing to higher EDIH scores [35]. TAGs are major components of very low-density lipoproteins (VLDL), and play an important role in storage of energy, storage of fatty acids, and provision of precursors for phospholipid biosynthesis [35]. DAGs consist of an ester derived from two long-chain fatty acids. Though DAGs are minor components of cell membranes, they are important intermediates in lipid metabolism and key elements in cell signaling [36]. Several studies have found adherence to higher dietary quality to be favorably associated with TAG concentrations [37,38,39] in line with the current study findings. Another mechanistic aspect relates to mitochondrial fatty acid oxidation. Mitochondrial dysfunction resulting in lower oxidative capacity may lead to higher accumulation of intracellular lipids which interfere with insulin signaling, leading to insulin resistance [40,41]. Given that acylcarnitine profiling has been shown to be predictive of mitochondrial fatty acid oxidation defects [42,43], it is possible that higher insulinemic potential of the diet, which correlates with higher acylcarnitines abundances, may influence health outcomes through impaired mitochondrial fatty acid oxidation.

Previous studies [15,44,45,46] have investigated differences in metabolomic profiles among people consuming a variety of other dietary patterns. For example, a previous study examined differences in the abundances of 118 metabolites among meat eaters, fish eaters, vegetarians and vegans, and found that 79% of metabolites significantly differed by diet group. In the majority of these metabolites, meat eaters most often had the highest concentrations, whereas vegans had the lowest concentration of acylcarnitines, sphingolipids and glycerophospholipids; and vegetarians or fish eaters had the highest concentrations of amino acids. The metabolic profiles of vegans was clearly separated from that of the other diet groups, with vegans having lower concentrations of some glycerophospholipids similar to the current study [44]. Another study examined the correlation of four diet quality indices [the Healthy Eating Index (HEI) 2010, the Alternate Mediterranean Diet Score (aMED), the WHO Healthy Diet Indicator (HDI), and the Baltic Sea Diet (BSD)] with serum metabolites [15], and found that the HEI-2010, aMED, HDI, and BSD were associated with 23, 46, 23, and 33 metabolites, respectively. Additionally, the dietary indices were associated with metabolites correlated with most components used to score adherence to the dietary pattern [15]. While specific metabolites may not always be reproduced across different studies due to differences in metabolomic platforms, categories of metabolites may align between studies in terms of diet quality more broadly. For example, a low-quality dietary pattern (high EDIH) may be found to be associated with high concentrations of different acylcarnitines in different studies. 

Associations appeared to differ by body weight. Twelve metabolites were associated with EDIH among normal weight women at the FDR adjusted *P* < 0.05, whereas up to 64 metabolites were associated with EDIH among overweight or obese women at the FDR adjusted *P* < 0.05, including 10 of the 12 metabolites among normal weight women, though the magnitudes of association among normal weight women for these 10 metabolites were stronger. Among the 64 metabolites observed in overweight and obese women, all five cholesterol esters and all 22 phospholipids or lysophospholipids were associated with lower dietary insulinemic potential whereas all six acyl carnitines and all 10 TAGs and DAGs were associated with higher dietary insulinemic potential. Lysophospholipids are signaling molecules involved in modulating processes such as inflammation, insulin secretion and insulin sensitivity through their interaction with G protein-coupled receptors [47,48]. Lysophospholipids may therefore be important in obesity and related diseases. A study assessing plasma lysophospholipids in obesity found that a combination of 26 lysophospholipids could discriminate between normal weight and obese participants with an accuracy of 98% [49]. Among the obese participants, the authors in that study, found decreased concentrations of different lysophospholipid species including lysophosphatidylcholines, and lysophosphatidylethanolamines [49]. These results are consistent with those observed in the current study. 

Our study has several strengths, including a validated metabolomics platform, detailed covariate data, and a robust methodology. Notably, our methodology includes the integration of dietary data with metabolomics to characterize the metabolomics profiles of insulinemic diets. Limitations of our study include known measurement error in using an FFQ for the assessment of diet, such as underreporting of energy and protein intake [50,51]. Given that energy misreporting may depend on BMI, we adjusted all models for BMI including the adjustment of BMI subgroup analysis for continuous BMI. Our findings may also have limited generalizability, therefore additional data are needed to identify whether these results are specific to postmenopausal women or whether there are similar metabolic profiles in men or in premenopausal women. In addition, we had limited sample sizes in the BMI subgroup analyses and could only explore statistical replication of the metabolites found, but we note that 14 of the 19 metabolites replicated in the primary analyses were also replicated in these exploratory analyses at an FDR-adjusted *P* < 0.05 among overweight/obese women. In addition, given current eating patterns of multiple regular meals and snacks, which lead to a perpetual postprandial state for the majority of the day; it is ideal to have multiple measures of insulin or c-peptide concentrations over the course of a day for each participant. However, we have previously shown that habitual diets predict a pattern of c-peptide concentrations that approximates the expected physiologic pattern and a single blood sample may therefore provide an unbiased mean estimate of c-peptide concentration for the population [2].

In summary, in two independent datasets of postmenopausal women, multiple metabolites associated with the insulinemic potential of diet were identified and statistically replicated. We found that metabolites involved in the metabolic pathways of cholesteryl esters, phospholipids, acylglycerols and acylcarnitines may provide insights on the biological mechanisms through which insulinemic dietary patterns influence disease risk and outcomes. The linkage of these findings to disease requires further study.

## 4. Methods

### 4.1. Study Population

The Women’s Health Initiative (WHI) enrolled 161,808 postmenopausal women 50 to 79 years old in 40 sites in the United States between 1993 and 1998 [52]. Participants were enrolled into an observational study (OS) or one or more of four clinical trials, two of which were hormone therapy (HT) trials. One of the HT trials randomly assigned 10,739 women with prior hysterectomy, to estrogen or placebo, whereas the other randomized 16,608 women to estrogen plus progestin or placebo. The full WHI-OS consisted of 93,676 women not eligible or unwilling to participate in the clinical trials [52]. At the baseline clinic visit, trained study nurses drew blood samples and performed physical measurements including blood pressure, height and weight. 

For the EDIH validation study, using fasting plasma c-peptide data; we pooled data from two nested case-control studies that measured plasma c-peptide at WHI baseline. The two studies comprised 1005 women. We excluded women with very low or very high body mass index (BMI) values (<15 kg/m^2^ or >50 kg/m^2^), those with implausible total energy intake values (≤600 kcal/d or ≥5000 kcal/d), and those who self-reported diabetes at the baseline. After the exclusions, 919 women were retained for analyses in the EDIH validation study. 

For the EDIH metabolomics study; we used data from 2306 participants from the Metabolomics of CHD in the WHI study [6], a matched case-control study that selected participants from the OS and HT. The cases (who developed coronary heart disease (CHD) after the baseline fasting blood draw) were frequency matched to controls on race/ethnicity, hysterectomy status, 5-year age groups, and 2-year enrollment window. After exclusions, the analytic dataset included 1919 women: 1109 in the WHI-HT (discovery dataset) and 810 in the WHI-OS (replication dataset). 

There was very minimal overlap (*n* = 15) between participants with c-peptide data, and the participants in the Metabolomics of CHD in the WHI study. Therefore, we conducted analyses using separate study populations for EDIH construct validation and EDIH metabolomics. The WHI protocol was approved by the institutional review boards at the Clinical Coordinating Center at the Fred Hutchinson Cancer Research Center in Seattle, WA, and at each of the 40 Clinical Centers [52]. The current study was approved by the institutional review board at the Brigham and Women’s Hospital (IRB protocol # 2013P000568).

### 4.2. Dietary Assessment and Calculation of the Empirical Dietary Index for Hyperinsulinemia (EDIH) Score

At the baseline, all WHI participants completed a 122-item food frequency questionnaire (FFQ) by self-report, developed for the WHI to estimate average daily dietary intake over the previous 3-month period [52,53]. The WHI FFQ has produced results reasonably comparable to those from four 24-h dietary recall interviews and four days of food diaries recorded within the WHI [51,54].

The development and validation of the EDIH score has been described in detail elsewhere [1]. Briefly, the score was developed in a sample of women in the Nurses’ Health Study (NHS), to empirically create a score to assess the insulinemic potential of whole diets defined using foods. Thirty-nine foods and food groups (servings/day) [55] were entered into stepwise linear regression analyses to identify a dietary pattern most predictive of c-peptide concentrations. The EDIH score is a weighted sum of 18 food groups, with higher scores reflective of higher insulinemic potential of the diet (hyperinsulinemia) and lower scores indicating lower insulinemic diets. The food groups contributing to high EDIH scores include: Red meat, low-energy beverages, cream soups, processed meat, margarine, poultry, French fries, non-dark (non-oily) fish, high energy beverages, tomatoes, low-fat dairy and eggs. The food groups contributing to lower EDIH score include: Whole fruit, wine, coffee, high-fat dairy products and green leafy vegetables [1]. 

In the NHS, the Spearman correlation coefficient between EDIH and c-peptide levels was 0.21; and there was a 40% increase in c-peptide concentration among the women in EDIH quintile 5 compared to those in quintile 1 (adjusted relative concentration: 1.40; 95% CI, 1.32, 1.46) [1]. The EDIH score was evaluated in two independent U.S.-based cohorts of women (NHS-II, *n* = 1,717) and men-Health Professionals Follow-up Study (HPFS, *n* = 4002) and found to significantly predict c-peptide concentrations. For example, the adjusted relative concentration of c-peptide in the highest quintile compared to the lowest was 1.29 (95% CI, 1.22, 1.39) in the HPFS and 1.32 (95% CI, 1.21, 145) in the NHS-II [1]. In these validation studies, the correlation coefficients were 0.14 (men) and 0.20 (women) [1]. In the current study, we calculated EDIH scores for each participant based on self-administered FFQ data collected at WHI baseline.

### 4.3. C-peptide Measurement

Blood was drawn from WHI participants at the first screening visit (1994–1998). All blood samples were continuously stored in well-monitored liquid nitrogen freezers from blood collection to their retrieval for analysis. Details on blood draw, transportation, and storage in these cohorts have been described [56]. Procedures for the measurement of plasma c-peptide concentrations in the WHI have also been described [57]. Briefly, plasma c-peptide was measured using ELISA reagents (Diagnostic Systems Laboratories/Beckman Coulter, Webster, TX, USA). In the nested case-control studies in which c-peptide was measured, samples from cases (that developed after the baseline blood collection) and their matched controls were analyzed in the same batch. Quality control samples were randomly interspersed among the case-control samples, and laboratory personnel were blinded to quality control and case-control status for all assays. The average intra-assay coefficients of variation from the internal quality control samples were 3.02% and 2.65% for the two nested case-control studies.

### 4.4. Assessment of Metabolites 

Plasma samples for the metabolomics study were collected and processed as described for the validation study. Metabolomic measurements were conducted at the Broad Institute (Boston, MA, USA), using four complimentary liquid chromatography tandem mass spectroscopy (LC-MS) methods, described in detail elsewhere [6], resulting in 509 metabolites. For each method, pooled plasma reference samples were included after running every 20 samples, and results were standardized using the ratio of the value of the sample to the value of the nearest pooled reference multiplied by the median of all reference values for the metabolite [6]. All signals were inspected to ensure quality and integration, and a signal-to-noise ratio <10 was considered unquantifiable [58,59]. For each method, metabolite identities were confirmed using authentic reference standards or reference samples. Coefficients of variation (CVs) were calculated using pooled plasma samples from the first 800 WHI-OS participants [6]. After excluding metabolites with a CV >20%, 448 known metabolites were retained for analyses. In the pilot testing of the metabolomics platform, 92% of metabolites had acceptable assay reproducibility (CV < 20%) and almost 90% of metabolites were stable over one to two years (Spearman correlation or ICC ≥ 0.4) [60].

### 4.5. Covariates

Data on covariates were collected by self-administered questionnaires on medical history, demographics, and lifestyle factors at the baseline, as previously described [52]. Covariates adjusted for, were the following: Total energy intake (kcal/day); body mass index [BMI = weight (kg)/(height (m) × height (m))]; age at WHI baseline (years); racial/ethnic groups (American Indian or Alaskan Native, Asian or Pacific Islander, Hispanic/Latino, African American, European American, and other race groups); smoking status (current, former, and never); educational levels categorized into some high school or lower educational level, high school graduate or some college or associate degree, and ≥4 years of college; regular use of aspirin and other nonsteroidal anti-inflammatory drugs (NSAID) (yes/no); where regular use of medications was defined as: ≥2 times in each of the two weeks preceding the interview; category and duration of estrogen use and category and duration of combined estrogen and progestin use categorized into five groups (none, ≤4.9 y, 5–10.0 y, 10.1–14.9 y, and ≥15.0 y); self-reported recreational physical activity, calculated by summing the metabolic equivalent tasks for all reported activities for each individual (e.g., walking, aerobics, jogging, tennis, swimming, biking outdoors, exercise machine, calisthenics, popular or folk dancing) (MET-hours/week) [61]; hormone therapy trial arm (estrogen-alone intervention, estrogen-alone control, combined estrogen and progestin intervention, estrogen and progestin control, not randomized to trial); and calcium and vitamin D arm (intervention, control, not randomized to trial), CHD case-control status, dietary modification trial arm (intervention, control, not randomized to the trial). These covariates were included in both the validation study and metabolomics study. 

### 4.6. Statistical Analysis

We normalized the distributions of c-peptide and metabolites data by transforming them to the natural log scale. Missing metabolite values below the limit of detection were assigned to half the lowest observed value. We described participants’ characteristics using means (standard deviations) for continuous variables, and frequencies (%) for categorical variables; across quintiles of the EDIH. 

For the EDIH validation analysis in the WHI, we used multivariable-adjusted linear regression models to estimate the absolute and relative concentrations of plasma c-peptide in quintiles of the EDIH. We stratified the association between EDIH score and c-peptide by BMI (normal weight, overweight/obese women) and assessed potential effect modification using the Wald *p* value of the EDIH x BMI interaction term. 

For the discovery analysis in the larger WHI-HT dataset (*n* = 1109), each metabolite was evaluated individually in multivariable-adjusted linear regression models in relation to 5-SD increments in the EDIH score. All models included BMI and the other factors listed above. Statistical significance was based on a two-sided *P* < 0.05 and a corresponding false discovery rate-adjusted *P*-value of 0.05. Metabolites discovered in WHI-HT were evaluated individually in the independent WHI-OS dataset (*n* = 810) using the same models as in the discovery analysis. Metabolite associations were considered to have been replicated based on a two-sided *P* < 0.05 and a corresponding false discovery rate-adjusted *p* value of 0.05. In all analyses, the EDIH score was modelled as a continuous variable. 

In the secondary analyses, we conducted subgroup analysis in BMI categories: Normal weight (15 to <25 kg/m^2^), overweight/obese (25 to ≤50 kg/m^2^), while adjusting for continuous BMI within BMI categories. Due to power considerations, stratified analyses were conducted in the combined WHI-OS and WHI-HT datasets. Statistical significance was based on a two-sided *P* < 0.05 and a corresponding false discovery rate-adjusted *P*-value of 0.05. Additionally, and even though statistical power was limited, we conducted exploratory analyses in the BMI subgroups in separate discovery and replication datasets, similar to the primary analyses but at an FDR-adjusted *P* < 0.20 for statistical significance. 

## Figures and Tables

**Table 1:** Characteristics of the empirical dietary index for hyperinsulinemia (EDIH) score validation study population, *n* = 919 ^1,2^.

Characteristic	Quintile 1 (−4.44 to <−0.81), *n* = 183	Quintile 2 (–0.81 to <–0.31), *n* = 184	Quintile 3 (–0.31 to <0.07), *n* = 184	Quintile 4 (0.07 to <0.66), *n* = 184	Quintile 5 (0.66 to 4.93), *n* = 184
C-peptide, ng/mL	1.14 ± 0.80	1.20 ± 0.77	1.32 ± 0.76	1.36 ± 0.72	1.54 ± 0.78
Age at screening, years	66.7 ± 6.9	66.9 ± 6.7	67.1 ± 6.5	67.1 ± 6.6	65.3 ± 6.6
Body mass index, kg/m^2^	26.1 ± 4.5	26.6 ± 4.9	27.2 ± 5.5	28.0 ± 5.4	29.0 ± 5.7
Body mass index categories, %					
15–<18.5 (thin)	1.1	1.1	1.1	1.1	0
18.5–<25 (normal weight)	46.4	39.7	40.2	29.3	25.0
25–<30 (overweight)	36.6	39.7	34.2	44.6	38.0
30–50 (obese)	15.9	19.5	24.5	25.0	37.0
Physical activity, MET-hour/week	10.0 ± 11.7	9.4 ± 12.3	9.1 ± 11.0	7.3 ± 10.1	5.3 ± 7.7
Aspirin/NSAID user, %	53	56	57.1	53.3	52.3
Educational level, %					
Some high school or lower educational level	2.7	3.8	3.3	5.4	6.5
High school graduate/some college or associate degree	45.9	57.1	48.4	65.2	69.6
≥4y of college	51.4	39.1	48.4	29.4	23.9
Race/ethnicity, %					
African American	6.0	6.5	8.7	9.2	13.0
European American	89.6	86.4	84.2	89.1	79.4
Other	4.4	7.1	7.1	1.7	7.6
Smoking status, %					
Never	42.6	50.5	51.1	56.0	48.4
Former	51.9	43.5	44.0	37.5	42.4
Current	5.5	6.0	4.9	6.5	9.2
Menopausal hormone use, %					
Unopposed estrogen use, ever	32.8	39.7	37.0	42.9	34.8
Estrogen plus progestin use, ever	29.0	26.6	18.5	19.0	21.7

EDIH, empirical dietary index for hyperinsulinemia score; ^1^ Values are percentages or means ± standard deviations. ^2^ EDIH scores were adjusted for total energy intake using the residual method. Lower EDIH scores indicate low insulinemic diets whereas higher scores indicate hyperinsulinemic diets. ^3^ The EDIH score was calculated from the following food group (servings per day): Foods that contributed to higher c-peptide concentrations were: Red meat, high-energy sugary beverages (the WHI FFQ did not assess low-energy beverages separately from other sugary beverages), cream soup, processed meat, butter, margarine, poultry, French fries, non-dark or non-oily fish, low-fat dairy, eggs. Foods that contributed to lower c-peptide concentrations were: Wine, coffee or tea, fruits, high-fat dairy, and green leafy vegetables.

**Table 2:** Adjusted absolute and relative concentrations (95% CI) of plasma c-peptide in quintiles of the empirical dietary index for the hyperinsulinemia score; Women’s Health Initiative (*n* = 919) ^1,2,3^.

Statistical Models	EDIH Quintiles					*P-*Trend ^4^
	Quintile 1	Quintile 2	Quintile 3	Quintile 4	Quintile 5	
Absolute concentrations (ng/mL)						
Model 1	1.14 (1.07, 1.22)	1.20 (1.12, 1.28)	1.33 (1.24, 1.42)	1.37 (1.28, 1.46)	1.54 (1.44, 1.64)	<0.0001
Model 2	1.21 (0.94, 1.56)	1.26 (0.99, 1.61)	1.40 (1.10, 1.80)	1.37 (1.08, 1.76)	1.53 (1.19, 1.96)	<0.0001
Model 3	1.19 (0.95, 1.50)	1.22 (0.98, 1.53)	1.30 (1.04, 1.63)	1.41 (1.04, 1.63)	1.41 (1.13, 1.77)	<0.0001
Relative concentrations (percent change)						
Model 1	0 (ref)	5 (−7, 18)	16 (3, 31)	20 (6, 35)	34 (19, 51)	<0.0001
Model 2	0 (ref)	4 (−7, 17)	16 (3, 30)	13 (1, 28)	26 (12, 42)	<0.0001
Model 3	0 (ref)	3 (−8, 14)	12 (1, 25)	9 (−2, 22)	18 (6, 32)	<0.0001
Normal weight (BMI: 15 to <25 kg/m^2^, n = 340): absolute concentrations (ng/mL)						
Model 1 + BMI	0.98 (0.89, 1.05)	0.94 (0.87, 1.03)	1.14 (1.04, 1.24)	1.07 (0.97, 1.18)	1.09 (0.97, 1.22)	0.02
Model 3	0.90 (0.67, 1.21)	0.87 (0.65, 1.17)	1.09 (0.73, 1.32)	0.98 (0.73, 1.32)	0.97 (0.71, 1.32)	0.09
Normal weight (BMI: 15 to <25 kg/m^2^, *n* = 340): relative concentrations (percent change)						
Model 1 + BMI	0 (ref)	−2 (−16, 13)	18 (1, 37)	10 (−6, 30)	12 (−5, 34)	0.02
Model 3	0 (ref)	−3 (−17, 14)	21 (3, 42)	9 (−8, 30)	8 (−11, 30)	0.09
Overweight/obese (BMI: 25 to 50 kg/m^2^, *n* = 579): absolute concentrations (ng/mL)						
Model 1 + BMI	1.37 (1.26, 1.50)	1.44 (1.33, 1.57)	1.47 (1.36, 1.60)	1.51 (1.40, 1.63)	1.68 (1.56, 1.80)	0.008
Model 3	1.48 (1.10, 1.99)	1.55 (1.16, 2.08)	1.59 (1.19, 2.13)	1.60 (1.20, 2.13)	1.82 (1.36, 2.42)	0.0005
Overweight/obese (BMI: 25 to 50 kg/m^2^, *n* = 579): relative concentrations (percent change)						
Model 1 + BMI	0 (ref)	5 (−9, 22)	7 (−7, 25)	10 (−6, 27)	22 (6, 41)	0.008
Model 3	0 (ref)	5 (−10, 22)	8 (−7, 25)	8 (−7, 25)	23 (6, 42)	0.0005

^1^ Values are absolute back-transformed (ex) c-peptide concentrations since data were LN-transformed prior to the analyses. ^2^ Women who reported diabetes at the baseline (*n* = 56) were excluded from all analyses. Model 1 was adjusted for age at screening; model 2 was adjusted for covariates in model 1 and for physical activity, educational level, race/ethnicity, income, non-steroidal anti-inflammatory drug use, statin use, smoking status, duration of postmenopausal hormone use (separately for unopposed estrogen, combined estrogen and progestin), high cholesterol, hypertension, colitis, arthritis, dietary modification trial arm, hormone therapy trial arm, calcium and vitamin D trial arm; model 3 included all covariates in model 2 and body mass index (continuous). C-peptide concentrations were calculated at the mean values of the continuous covariates and at the reference category of the categorical covariates. ^3^ Wald *p* value for the interaction term was 0.02 between EDIH and BMI. ^4^ The *p*-value for the linear trend across EDIH quintiles was the *p*-value of the ordinal variable constructed by assigning quintile medians to all participants in the quintile. Models for the linear trend were adjusted for all covariates listed in the corresponding model.

**Table 3:** Distribution of dietary intakes across quintiles of the EDIH score (metabolomics study samples—combined discovery and replication datasets) ^1,2,3^.

-	Quintile 1 (–5.36 to <–0.72) *n* = 383	Quintile 2 (–0.72 to <–0.21) *n* = 384	Quintile 3 (–0.21 to <0.20) *n* = 384	Quintile 4 (0.20 to <0.74) *n* = 384	Quintile 5 (0.74 to 6.64) *n* = 384
Food/food groups, servings/week					
Red meat	3.3 ± 3.2	3.0 ± 2.8	3.2 ± 3.1	3.4 ± 2.7	4.8 ± 4.2
Sugar-sweetened beverages	0.4 ± 1.0	0.5 ± 1.4	0.5 ± 1.3	1.3 ± 2.8	4.3 ± 9.0
Cream soup	0.2 ± 0.4	0.2 ± 0.3	0.3 ± 0.4	0.3 ± 0.4	0.5 ± 0.8
Processed meat	1.2 ± 1.5	1.3 ± 1.5	1.7 ± 1.7	1.9 ± 2.1	3.7 ± 3.5
Butter and margarine	3.0 ± 3.6	3.3 ± 3.8	4.5 ± 4.3	6.0 ± 4.8	10.5 ± 9.0
Poultry	2.3 ± 1.7	2.3 ± 1.7	2.4 ± 1.8	2.5 ± 1.8	3.2 ± 2.4
White/non-oily fish	1.6 ± 1.5	1.4 ± 1.3	1.4 ± 1.2	1.5 ± 1.6	1.7 ± 1.8
French fries	0.2 ± 0.3	0.2 ± 0.3	0.2 ± 0.4	0.3 ± 0.5	0.7 ± 1.1
Tomatoes	3.6 ± 3.2	3.7 ± 3.6	3.2 ± 3.0	3.7 ± 3.6	4.4 ± 4.8
Low-fat dairy	14.8 ± 12.5	13.4 ± 11.8	14.2 ± 13.2	12.3 ± 11.9	13.5 ± 13.8
Eggs	0.7 ± 1.0	0.8 ± 1.4	0.9 ± 1.1	1.0 ± 1.1	1.6 ± 2.3
Refined grains	25.2 ± 14.5	21.4 ± 12.2	20.2 ± 12.7	19.8 ± 11.8	24.4 ± 14.4
Whole grains	9.9 ± 6.5	8.3 ± 5.3	7.6 ± 5.1	6.7 ± 4.6	7.3 ± 5.6
Wine	3.9 ± 6.0	1.1 ± 2.1	0.6 ± 1.3	0.5 ± 1.4	0.3 ± 1.0
Tea/coffee	21.1 ± 15.2	16.4 ± 12.0	14.0 ± 12.4	12.9 ± 11.7	13.0 ± 12.5
Whole fruit	18.3 ± 10.4	16 ± 8.8	13.0 ± 7.6	10.0 ± 7.0	9.4 ± 7.3
High-fat dairy	3.2 ± 4.1	2.3 ± 3.4	2.3 ± 3.1	2.1 ± 2.4	2.8 ± 3.3
Green-leafy vegetables	7.8 ± 6.1	6.3 ±4.5	5.7 ± 4.	5.0 ± 4.5	4.7 ± 4.0
Nutrient intakes					
Fiber, g/d	19.8 ± 7.6	16.8 ± 6.2	14.7 ± 5.6	12.8 ± 5.5	13.6 ± 6.3
Carbohydrate, g/d	235 ± 83	202 ± 66	185 ± 70	170 ± 65	204 ± 93
Protein, g/d	72.9 ± 29.0	64.1 ± 26.3	62.5 ± 28.5	59.7 ± 24.7	72.0 ± 32.9
Total fat, g/d	58.5 ± 29.0	50.8 ± 28.4	53.7 ± 30.2	56.1 ± 28.0	75.6 ± 41.7
Saturated fat, g/d	19.8 ± 10.7	17.0 ± 9.9	18.0 ± 10.9	18.6 ± 10.0	25.3 ± 15.0
Cholesterol, g/d	201 ± 119	191 ± 130	198 ± 119	207 ± 109	286 ± 191
Calcium, mg/d	978 ± 496	817 ± 413	780 ± 469	671 ± 380	737 ± 425
Lycopene, mcg/d	5539 ± 3657	5125 ± 3246	4164 ± 2557	4389 ± 3466	4651 ± 3465

EDIH, empirical dietary index for the hyperinsulinemia score; ^1^ Values are means ± standard deviations. ^2^ EDIH scores were adjusted for total energy intake using the residual method. Lower EDIH scores indicate low insulinemic diets whereas higher scores indicate hyperinsulinemic diets. ^3^ The EDIH component foods (servings per day) in the WHI were the following: Red meat (ground meat including hamburgers, beef, pork and lamb as a main dish, or as a sandwich; stew, pot pie and casseroles with meat; gravies made with meat drippings); high-energy sugary beverages, (all regular - not diet - soft drinks); low-energy sugary beverages (the WHI FFQ did not assess low-energy beverages separately from other sugar-sweetened beverages); cream soup (such as chowders, potato, tomato, cheese, ajiaco); processed meat (hot dogs, chorizo; other sausage, bacon, breakfast sausage, scrapple; lunch meat such as ham, turkey; other lunch meat such as bologna); butter, margarine (butter, margarine or oil, on bread or tortillas; margarine or butter added to cooked cereal or grits; butter, margarine, sour cream, oils, or other fat added to vegetables, beans, rice, and potatoes, after cooking); poultry (poultry); French fries (French fries, fried potatoes, fried rice, fried cassava and fritters); non-dark or non-oily fish (fried fish, shrimp, lobster, crab and oysters, canned tuna, tuna salad, and tuna casserole, white fish such as sole, snapper, cod); tomatoes (fresh tomato, tomato juice, tomato sauce, cooked tomato, salsa and salsa picante); low-fat dairy (part-skim or reduced fat cheeses, such as Mexican-type cheeses or mozzarella. Include cheese added to foods and in cooking; low-fat cottage cheese; low-fat or no-fat frozen desserts, such as frozen yogurt, sherbet, ice milk, and low-fat milkshakes; non-fat yogurt (not frozen); all other yogurt (not frozen); low-fat milk; Milk, cream, or creamer in coffee or tea); eggs (eggs); wine (red wine, white wine); coffee or tea (all types); fruits (all types); high-fat dairy (whole milk, evaporated/condense milk, ice cream, cottage cheese and ricotta cheese, other cheese); green leafy vegetables (cooked greens such as spinach, mustard greens, turnip greens, collards; lettuce and plain lettuce salad; mixed lettuce or spinach salad with vegetables).

**Table 4:** Associations of the empirical dietary index for the hyperinsulinemia score with metabolites in the discovery of Women’s Health Initiative-Hormone Therapy (WHI-HT) and replication of WHI- Observational Study (WHI-OS) datasets ^1,2,3^.

-	-	-	Associations in WHI-HT (Discovery, *n* = 1109)		Associations in WHI-OS (Replication, *n* = 810)	
Metabolite	HMDB ID	Category	Beta Estimate (95% CI)	FDR-Adjusted *P*-value	Beta Estimate (95% CI)	FDR-Adjusted *P*-value
C14:0 CE	HMDB0006725	Cholesterol esters	−0.57 (−0.87, −0.27)	0.015	−0.63 (−0.96, −0.30)	**1.83 × 10^4^**
C16:1 CE	HMDB0000658	Cholesterol esters	−0.63 (−0.91, −0.33)	0.008	−0.88 (−1.24, −0.52)	**6.26 × 10^6^**
C18:1 CE	HMDB0000918	Cholesterol esters	−0.50 (−0.78, −0.21)	0.018	−0.46 (−0.79, −0.12)	**0.009**
C18:3 CE	HMDB0010370	Cholesterol esters	−0.49 (−0.78, −0.20)	0.018	−0.41 (−0.76, −0.05)	**0.026**
C20:3 CE	HMDB0006736	Cholesterol esters	−0.49 (−0.78, −0.21)	0.018	−0.43 (−0.77, −0.08)	**0.016**
C20:5 CE	HMDB0006731	Cholesterol esters	−0.48 (−0.76, −0.19)	0.024	−0.46 (−0.83, −0.08)	**0.016**
Trigonelline	HMDB0000875	Alkaloid and derivatives	−0.54 (−0.82, −0.25)	0.015	−0.61 (−0.97, −0.27)	**5.14 × 10^4^**
C36:1 PS plasmalogen	Unknown	Other	−0.49 (−0.80, −0.18)	0.030	−0.69 (−1.03, −0.35)	**8.93 × 10^5^**
Eicosapentaenoate	HMDB0001999	Fatty acids	−0.47 (−0.74, −0.19)	0.018	−0.37 (−0.72, −0.02)	**0.038**
Myristoleic acid	HMDB0002000	Fatty acids	0.43 (0.14, 0.73)	0.047	0.16 (−0.16, 0.49)	0.325
C4−OH carnitine	HMDB0013127	Acylcarnitines	0.40 (0.12, 0.68)	0.048	0.25 (−0.12, 0.61)	0.179
C10:2 carnitine	HMDB0013325	Acylcarnitines	0.47 (0.17, 0.77)	0.030	0.58 (0.24, 0.92)	**9.09 × 10^4^**
C18:2 SM	HMDB0012101	Sphingomyelins	0.42 (0.13, 0.71)	0.048	0.78 (0.43, 1.14)	**3.40 × 10^5^**
C36:3 DAG	HMDB0007219	Diacylglycerols	0.46 (0.16, 0.75)	0.030	0.51 (0.15, 0.86)	**0.005**
C36:4 DAG−A	HMDB0007248	Diacylglycerols	0.53 (0.23, 0.83)	0.018	0.68 (0.33, 1.03)	**1.62 × 10^4^**
C51:3 TAG	Unknown	Triacylglycerols	0.48 (0.18, 0.77)	0.030	0.62 (0.27, 0.97)	**4.78 × 10^4^**
C52:3 TAG	HMDB0005384	Triacylglycerols	0.47 (0.16, 0.77)	0.033	0.38 (0.05, 0.72)	**0.026**
C52:4 TAG	HMDB0005363	Triacylglycerols	0.58 (0.28, 0.88)	0.015	0.56 (0.20, 0.91)	**0.002**
C54:2 TAG	HMDB0005403	Triacylglycerols	0.44 (0.15, 0.73)	0.035	0.20 (−0.15, 0.55)	0.269
C54:3 TAG	HMDB0005405	Triacylglycerols	0.47 (0.17, 0.77)	0.030	0.35 (−0.01, 0.71)	**0.054**
C54:4 TAG	HMDB0005370	Triacylglycerols	0.53 (0.23, 0.84)	0.018	0.54 (0.17, 0.92)	**0.004**
C54:6 TAG	HMDB0005391	Triacylglycerols	0.55 (0.25, 0.86)	0.018	0.46 (0.10, 0.82)	**0.013**
cAMP	HMDB0000058	Purines and Pyrimidines	0.37 (0.12, 0.62)	0.047	0.20 (−0.68, 0.27)	0.401
N4-acetylcytidine	HMDB0005923	Purines and Pyrimidines	0.43 (0.16, 0.71)	0.030	0.10 (−0.24, 0.44)	0.563
Isoleucine	HMDB0000172	Amino acids	0.47 (0.20, 0.74)	0.018	0.13 (−0.23, 0.49)	0.472
Cystathionine	HMDB0000099	Amino Acids	0.51 (0.23, 0.79)	0.018	0.07 (−0.28, 0.42)	0.689

^1^ All values are beta estimates obtained from multivariable-adjusted linear regression modeling 5-unit increments of EDIH as the main predictor of interest and metabolite as the main response variable of interest. ^2^ Models were adjusted for body mass index (continuous) age, physical activity, educational level, race/ethnicity, aspirin/NSAIDs use, smoking status, WHI Hormone Therapy trial arm, and CHD case-control status. ^3^ Statistical significance was defined as false-discovery rate adjusted P < 0.05 (in addition, significant *p* values in the replication dataset are highlighted in bold font).

**Table 5:** Metabolite discovery among normal weight women (*n* = 630) ^1,2,3^.

Metabolite	HMDB ID	Category	Beta Estimate (95% CI)	FDR-Adjusted *P*-value
**C14:0 CE**	HMDB0006725	Cholesteryl esters	−0.75 (−1.15, −0.35)	**0.016**
**C16:1 CE**	HMDB0000658	Cholesteryl esters	−1.05 (−1.49, −0.61)	**0.001**
C20:5 CE	HMDB0006731	Cholesteryl esters	−0.65 (−1.09, −0.22)	0.057
N-acetylornithine	HMDB0003357	Other	−0.82 (−1.23, −0.42)	**0.006**
C22:6 LPE	HMDB0011526	Lysophosphatidylethanolamine	−0.56 (−0.98, −0.14)	0.097
C34:0 PS	HMDB0012356	Other	−0.68 (−1.12, −0.24)	0.053
C30:0 PC	HMDB0007869	Phosphatidylcholines	−0.60 (−1.02, −0.18)	0.079
C30:1 PC	HMDB0007870	Phosphatidylcholines	−0.53 (−0.93, −0.14)	0.097
C32:1 PC	HMDB0007873	Phosphatidylcholines	−0.85 (−1.27, −0.42)	**0.008**
C32:1 PC plasmalogen-A	HMDB0013404	Phosphatidylcholine plasmalogens	−0.53 (−0.92, −0.15)	0.095
C34:1 PC	HMDB0007972	Phosphatidylcholines	−0.77 (−1.19, −0.35)	**0.019**
**C36:1 PS plasmalogen**	Unavailable	Phosphatidylethanolamine plasmalogens	−0.68 (−1.10, −0.25)	**0.045**
C36:4 PE	HMDB0008937	Phosphatidylethanolamine	−0.58 (−1.01, −0.15)	0.097
C36:5 PC	HMDB0007890	Phosphatidylcholines	−0.76 (−1.20, −0.32)	**0.031**
1-methylguanosine	HMDB0001563	Purines and Pyrimidines	−0.61 (−1.02, −0.21)	0.057
Urate	HMDB0000289	Purines and Pyrimidines	−0.54 (−0.93, −0.15)	0.095
Palmitoleic acid	HMDB0003229	Fatty acids	−0.61 (−1.03, −0.19)	0.079
Myristoleic acid	HMDB0002000	Fatty acids	0.69 (0.27, 1.12)	**0.043**
C18:0 LPC plasmalogen	HMDB0011149	Lysophosphatidylcholine plasmalogens	0.55 (0.14, 0.97)	0.097
C18:1 LPC plasmalogen	HMDB0011149	Lysophosphatidylcholine plasmalogens	0.56 (0.14, 0.98)	0.097
**C18:2 SM**	HMDB0012101	Sphingomyelins	0.90 (0.50, 1.31)	**0.002**
C22:1 MAG	HMDB0011582	Monoacylglycerols	−0.60 (−1.01, −0.19)	0.076
**C36:4 DAG-A**	HMDB0007248	Diacylglycerols	0.68 (0.26, 1.11)	**0.043**
C51:3 TAG	Unavailable	Triacylglycerols	0.58 (0.17, 0.99)	0.085
C54:3 TAG	HMDB0005405	Triacylglycerols	0.55 (0.13, 0.98)	0.106
**C54:4 TAG**	HMDB0005370	Triacylglycerols	0.76 (0.31, 1.20)	**0.037**
**C54:6 TAG**	HMDB0005391	Triacylglycerols	0.70 (0.26, 1.15)	**0.050**
Trimethylamine-N-oxide	HMDB0000925	Other	0.56 (0.15, 0.98)	0.096
Glycoursodeoxycholate	HMDB0000708	Bile acids	0.58 (0.15, 1.02)	0.097

^1^ All values are beta estimates obtained from multivariable-adjusted linear regression modeling 5-unit increments of EDIH as the main predictor of interest and metabolite as the main response variable of interest. ^2^ Models were adjusted for body mass index (continuous) age, physical activity, educational level, race/ethnicity, aspirin/NSAIDs use, smoking status, WHI Hormone Therapy trial arm, and CHD case-control status. ^3^ Statistical significance was defined as false-discovery rate adjusted *P* < 0.05 (in addition, all 12 significant *p* values are highlighted in bold, including the 7 metabolites that are among the 19 replicated metabolites in the primary analysis).

**Table 6:** Metabolite discovery among overweight or obese women (*n* = 1289) ^1,2,3^.

Metabolite	HMDB ID	Category	Beta Estimate (95% CI)	FDR-Adjusted *P*-value
**Eicosapentaenoate**	HMDB0001999	Fatty acids	−0.65 (−0.91, −0.40)	7.63 × 10^5^
Palmitoleic acid	HMDB0003229	Fatty acids	−0.39 (−0.67, −0.12)	0.032
Myristoleic acid	HMDB0002000	Fatty acids	0.38 (−0.11, 0.64)	0.035
2−hydroxyhexadecanoate	HMDB0031057	Fatty acids	0.39 (0.12, 0.66)	0.032
**C14:0 CE**	HMDB0006725	Cholesterol esters	−0.54 (−0.81, −0.27)	0.003
**C16:1 CE**	HMDB0000658	Cholesterol esters	−0.61 (−0.87, −0.34)	5.93 × 10^4^
**C18:1 CE**	HMDB0000918	Cholesterol esters	−0.49 (−0.76, −0.22)	0.006
**C18:3 CE**	HMDB0010370	Cholesterol esters	−0.45 (−0.72, −0.18)	0.012
**C20:3 CE**	HMDB0006736	Cholesterol esters	−0.49 (−0.76, −0.22)	0.006
**C20:5 CE**	HMDB0006731	Cholesterol esters	−0.37 (−0.64, −0.10)	0.035
**Trigonelline**	HMDB0000875	Alkaloid and derivatives	−0.67 (−0.93, −0.41)	7.63 × 10^5^
C16:1 LPC	HMDB0010383	Phosphatidylcholines	−0.54 (−0.81, −0.26)	0.003
C20:1 LPC	HMDB0010391	Phosphatidylcholines	−0.54 (−0.82, −0.27)	0.003
C24:0 LPC	HMDB0008038	Phosphatidylcholines	−0.49 (−0.76, −0.23)	0.005
C28:0 PC	HMDB0007866	Phosphatidylcholines	−0.37 (−0.65, −0.10)	0.040
C30:0 PC	HMDB0007869	Phosphatidylcholines	−0.40 (−0.67, −0.13)	0.026
C30:1 PC	HMDB0007870	Phosphatidylcholines	−0.43 (−0.70, −0.17)	0.014
C32:1 PC	HMDB0007873	Phosphatidylcholines	−0.40 (−0.66, −0.13)	0.026
C34:1 PC	HMDB0007972	Phosphatidylcholines	−0.35 (−0.62, −0.08)	0.046
C40:10 PC	HMDB0008511	Phosphatidylcholines	−0.34 (−0.61, −0.08)	0.050
C32:1 PC plasmalogen-A	HMDB0013404	Phosphatidylcholine plasmalogens	−0.40 (−0.67, −0.13)	0.024
C34:2 PC plasmalogen-B	HMDB0011210	Phosphatidylcholine plasmalogens	−0.44 (−0.70, −0.18)	0.012
C14:0 LPC	HMDB0010379	Lysophosphatidylcholines	−0.39 (−0.66, −0.12)	0.032
C14:0 LPC-A	HMDB0010379	Lysophosphatidylcholines	−0.44 (−0.71, −0.17)	0.014
C18:1 LPC	HMDB0002815	Lysophosphatidylcholines	−0.36 (−0.63, −0.09)	0.042
C18:3 LPC	HMDB0010387	Lysophosphatidylcholines	−0.38 (−0.66, −0.10)	0.040
C20:3 LPC	HMDB0010393	Lysophosphatidylcholines	−0.37 (−0.64, −0.09)	0.040
C16:0 LPE	HMDB0011503	Lysophosphatidylethanolamines	−0.45 (−0.72, −0.18)	0.012
C18:1 LPE	HMDB0011506	Lysophosphatidylethanolamines	−0.38 (−0.66, −0.11)	0.036
C22:6 LPE-B	HMDB0011526	Lysophosphatidylethanolamines	−0.36 (−0.63, −0.09)	0.040
C14:0 SM	HMDB0012097	Sphingomyelins	−0.45 (−0.71, −0.18)	0.012
**C18:2 SM**	HMDB0012101	Sphingomyelins	0.39 (0.12, 0.66)	0.032
C24:1 SM	HMDB0012107	Sphingomyelins	−0.43 (−0.70, −0.16)	0.017
C4-OH carnitine	HMDB0013127	Acylcarnitines	0.43 (0.17, 0.68)	0.012
C6 carnitine	HMDB0000705	Acylcarnitines	0.47 (0.20, 0.74)	0.011
C7 carnitine	HMDB0013238	Acylcarnitines	0.47 (0.21, 0.72)	0.006
C9 carnitine	HMDB0013288	Acylcarnitines	0.43 (0.17, 0.70)	0.014
**C10:2 carnitine**	HMDB0013325	Acylcarnitines	0.60 (0.34, 0.87)	7.01 × 10^4^
C14:2 carnitine	HMDB0013331	Acylcarnitines	0.38 (0.11, 0.65)	0.032
**C36:3 DAG**	HMDB0007219	Diacylglycerols	0.46 (0.19, 0.73)	0.012
**C36:4 DAG-A**	HMDB0007248	Diacylglycerols	0.56 (0.29, 0.83)	0.002
**C51:3 TAG**	Unknown	Triacylglycerols	0.53 (0.26, 0.79)	0.003
C52:2 TAG	HMDB0005369	Triacylglycerols	0.34 (0.08, 0.60)	0.047
**C52:3 TAG**	HMDB0005384	Triacylglycerols	0.46 (0.19, 0.73)	0.011
**C52:4 TAG**	HMDB0005363	Triacylglycerols	0.59 (0.32, 0.86)	0.001
C54:2 TAG	HMDB0005403	Triacylglycerols	0.38 (0.12, 0.65)	0.032
**C54:3 TAG**	HMDB0005405	Triacylglycerols	0.37 (0.10, 0.64)	0.036
**C54:4 TAG**	HMDB0005370	Triacylglycerols	0.45 (0.18, 0.72)	0.012
**C54:6 TAG**	HMDB0005391	Triacylglycerols	0.45 (0.18, 0.72)	0.012
Isoleucine	HMDB0000172	Amino acids	0.47 (0.22, 0.72)	0.005
Dimethylglycine	HMDB0000092	Amino Acids	0.40 (0.12, 0.66)	0.032
Cystathionine	HMDB0000099	Amino Acids	0.33 (0.08, 0.58)	0.046
2-aminooctanoate	HMDB0000991	Amino Acids	0.38 (0.10, 0.66)	0.038
Pantothenate	HMDB0000210	Amino Acids	0.34 (0.61, 0.08)	0.047
N-methylproline	HMDB0094696	Amino Acids	0.44 (0.70, 0.17)	0.012
**C36:1 PS plasmalogen**	Unknown	Other	0.55 (0.83, 0.27)	0.003
X4-pyridoxate	Unknown	Other	0.42 (0.67, 0.16)	0.014
Proline betaine	HMDB0004827	Other	0.40 (0.66, 0.14)	0.024
Indole-3-propionate	HMDB0002302	Other	0.35 (0.61, 0.09)	0.040
Cortisol	HMDB0000063	Steroids	0.37 (0.64, 0.10)	0.040
C23:0 Ceramide (d18:1)	HMDB0000950	Ceramides	0.39 (0.12, 0.66)	0.032
N4-acetylcytidine	HMDB0005923	Purines and Pyrimidines	0.37 (0.11, 0.62)	0.032
Cytidine	HMDB0000089	Purines and Pyrimidines	0.37 (0.10, 0.64)	0.040

^1^ All values are beta estimates obtained from multivariable-adjusted linear regression modeling 5-unit increments of EDIH as the main predictor of interest and metabolite as the main response variable of interest. ^2^ Models were adjusted for body mass index (continuous) age, physical activity, educational level, race/ethnicity, aspirin/NSAIDs use, smoking status, WHI Hormone Therapy trial arm, and CHD case-control status. ^3^ Statistical significance was defined as false-discovery rate adjusted *P* < 0.05 (in addition, all 19 metabolites replicated in the primary analysis are highlighted in bold font).

## Supplementary Materials

The following supplementary tables are available online at https://www.mdpi.com/2218-1989/9/6/120/s1. Table S1: Baseline characteristics of the study population across EDIH quintiles in the discovery and replication datasets, Table S2: Thirty-seven metabolites were significantly associated with EDIH at the raw *P*-value < 0.05, and none at an FDR-adjusted *P* < 0.20, among normal weight women in the discovery dataset (Hormone Therapy trial: *n* = 317), Table S3: Metabolites associated with EDIH at an FDR-adjusted *P* < 0.20 in the discovery and replication datasets, among overweight or obese women.
