# Supplementary material for: Identifying Metabolomic Profiles of Insulinemic Dietary Patterns
Source: Metabolites. 2019 Jun 24;9(6):120. doi: 10.3390/metabo9060120 (PMC6630814; doi:10.3390/metabo9060120)
Supplement: Supplementary file 1 [file metabolites-09-00120-s001.pdf]

Table S1. Baseline characteristics of the study population across EDIH quintiles in the discovery and replication datasets[1,2]

Characteristic	WHI-HT - discovery (n=1109)					WHI-OS - Validation (n=810)				
	Quintile 1 (-5.05, < -0.60) n=221	Quintile 2 (-0.60, < -0.14) n=222	Quintile 3 (-0.14, <0.30) n=222	Quintile 4 (0.30, <0.86) n=222	Quintile 5 (0.86, 6.64) n=222	Quintile 1 (-5.36, < -0.80) n=162	Quintile 2 (-0.80, < -0.33) n=162	Quintile 3 (-0.33, < 0.05) n=162	Quintile 4 (0.05, <0.58) n=162	Quintile 5 (0.58, 4.84) n=162
Age, years	67.9 ± 6.8	67.1 ± 6.7	67.2 ± 7.0	66.4 ± 6.9	65.9 ± 7.2	67.5 ± 6.2	68.5 ± 6.2	68.0 ± 6.7	67.3 ± 6.9	66.5 ± 7.5
Fasting glucose, mg/dL	96.1 ± 21.7	97.7 ±21.6	96.5 ± 15.7	100.6 ± 23.4	103.8 ± 29.8	91.9 ± 15.7	93.8 ± 12.7	96.2 ± 16.7	98.0 ± 17.7	103.0 ± 36.0
HDL cholesterol, mg/dL	53.5 ± 13.8	51.3 ±12.1	49.8 ± 12.1	47.9 ± 12.1	48.8 ± 12.3	57.3 ± 18.0	56.5 ± 16.6	55.2 ± 15.5	54.2 ± 16.6	51.4 ± 15.6
LDL cholesterol, mg/dL	156.1 ± 35.9	152.9 ± 35.3	159.6 ± 32.9	157.4 ± 37.4	157.9 ± 40.1	117.3 ± 38.2	129.7 ± 45.1	135.3 ± 35.9	136.3 ± 42.7	140.6 ± 43.2
Total cholesterol, mg/dL	236.1 ± 39.9	233.3 ± 38.4	241.2 ± 36.8	239.5 ± 42.4	238.5 ± 44.9	237.3 ± 47.7	232.3 ± 45.5	230.0 ± 43.8	232.6 ± 48.8	234.9 ± 49.5
Triglycerides, mg/dL	133.3 ± 65.9	144.6 ± 77.4	158.8 ± 87.9	170.3 ± 92.9	156.5 ± 80.3	139.2 ± 83.1	130.5 ± 87.2	130.6 ± 65.8	145.8 ± 79.4	136.2 ± 61.7
BMI, kg/m^2	26.6 ± 5.0	28.7 ± 5.4	28.3 ± 5.2	29.9 ± 5.8	30.0 ± 5.7	26.4 ± 5.3	25.9 ± 4.7	27.1 ± 5.0	27.3 ± 5.3	28.9 ± 5.8
Body mass index categories, %										
15 - < 18.5 (thin)	1.8	0.4	0.9	0.4	0.9	1.2	3.7	0.6	0.6	0
18.5 - < 25 (normal weight)	40.3	25.7	29.3	22.5	20.7	43.2	46.9	37.0	34.0	25.9
25 - < 30 (overweight)	36.2	41.0	33.8	28.4	32.9	37.7	30.3	34.0	39.5	40.1
30 - 50 (obese)	21.7	32.9	36.0	48.7	45.5	17.9	19.1	28.4	25.9	34.0
Physical activity, MET-hours/week	7.8 ± 9.7	7.0 ± 11.1	6.3 ± 8.6	5.6 ± 8.8	4.2 ± 8.4	10.1 ± 11.7	9.4 ± 13.7	9.5 ± 11.9	5.7 ± 8.0	6.3 ± 10.7
Aspirin/NSAIDs use, %	15.4	19.4	18.0	13.5	16.7	13.6	16.7	12.4	13.0	14.8
Educational level, %										
Less than high school	5.9	5.9	9.5	9.9	9.9	3.7	4.9	6.2	5.6	10.5
High school/GED	52.5	61.7	66.7	69.8	72.1	53.7	55.6	61.1	66.7	60.5
≥4 years of college	41.6	32.4	23.9	20.3	18.0	42.6	39.5	32.7	27.8	29.0
Race/ethnicity										
Black	4.5	11.3	5.0	10.4	18.0	4.3	10.5	11.7	14.2	22.8

Hispanic	1.8	1.8	3.6	5.0	0.9	3.1	1.9	3.1	3.1	3.1
White	91.9	84.2	90.1	82.9	78.8	81.5	82.1	76.5	72.8	67.3
Other	1.8	2.7	1.4	1.8	2.3	11.1	5.6	8.6	9.9	6.8
Smoking, %										
Never	48.0	50.5	53.2	46.0	44.6	46.9	49.4	48.8	53.7	39.5
Former	40.3	39.2	33.3	37.8	36.0	46.3	46.3	45.1	38.9	45.7
Current	11.8	10.4	13.5	16.2	19.4	6.8	4.3	6.2	7.4	14.8

[1]Values are percentages or means ± SDs. EDIH, empirical dietary index for hyperinsulinemia score; MET-h, metabolic equivalent hours; NSAID, nonsteroidal anti-inflammatory drugs; CRP, C-reactive protein; WHI, Women's Health Initiative; HT, Hormone Therapy trial; OS, Observational Study.

[2]EDIH scores were adjusted for total energy intake using the residual method. Lower EDIH scores indicate low insulinemic diets whereas higher scores indicate hyperinsulinemic diets.

Table S2. Thirty-seven metabolites were significantly associated with EDIH at the raw P-value <0.05, and none at FDR-adjusted P<0.20, among normal weight women in the discovery dataset (Hormone Therapy trial: n=317)[1,2,3]

**Metabolite**	**HMDB ID**	**Beta estimate (95%CI)**	**Raw P-value**	**FDR-adjusted P-value**
**C14:0 CE**	HMDB0006725	-0.98 (-1.59, -0.37)	0.002	**0.147**
C16:0 CE	HMDB0000885	-0.59 (-1.15, -0.02)	0.042	0.439
**C16:1 CE**	HMDB0000658	-1.17 (-1.80, -0.54)	2.91 E-04	**0.052**
**C18:1 CE**	HMDB0000918	-0.55 (-1.08, -0.02)	0.040	0.439
**C18:3 CE**	HMDB0010370	-0.69 (-1.26, -0.11)	0.019	0.388
**C20:5 CE**	HMDB0006731	-0.68 (-1.26, -0.09)	0.023	0.388
C30:0 PC	HMDB0007869	-0.84 (-1.41, -0.26)	0.005	0.242
C32:1 PC	HMDB0007873	-0.88 (-1.42, -0.33)	0.002	**0.147**
C32:1 PC plasmalogen-B	HMDB0013404	-0.70 (-1.33, -0.08)	0.028	0.413
C32:1 PC plasmalogen-A	HMDB0013404	-0.59 (-1.16, -0.02)	0.042	0.439
C34:1 PC	HMDB0007972	-0.69 (-1.28, -0.10)	0.022	0.388
C36:5 PC	HMDB0007890	-0.78 (-1.34, -0.21)	0.007	0.326
C34:0 PS	HMDB0012356	-0.71 (-1.29, -0.12)	0.018	0.388
C34:0 PE	HMDB0008925	-0.55 (-1.09, -0.0004)	0.050	0.480
C36:4 PE	HMDB0008937	-0.59 (-1.14, -0.03)	0.038	0.439
Xanthine	HMDB0000292	-0.67 (-1.23, -0.11)	0.019	0.388
N-acetylornithine	HMDB0003357	-0.62 (-1.19, -0.06)	0.031	0.438
1-methylguanosine	HMDB0001563	-0.60 (-1.16, -0.05)	0.034	0.439
Docosahexaenoic acid	HMDB0002183	-0.79 (-1.39, -0.18)	0.011	0.378
Docosatrienoic acid	HMDB0002823	-0.78 (-1.40, -1.16)	0.014	0.388
Urate	HMDB0000289	-0.69 (-1.34, -0.03)	0.039	0.439
Palmitoleic_acid	HMDB0003229	-0.69 (-1.27, -0.11)	0.020	0.388
Arachidonate	HMDB0001043	-0.58 (-1.16, -0.002)	0.049	0.480
C22:1 MAG	HMDB0011582	-0.60 (-1.12, -0.07)	0.027	0.413
**C54:3 TAG**	HMDB0005405	0.82 (0.21, 1.43)	0.008	0.326

**C54:4 TAG**	HMDB0005370	0.95 (0.32, 1.58)	0.003	0.219
**C54:6 TAG**	HMDB0005391	0.77 (0.12, 1.41)	0.020	0.388
**Myristoleic acid**	HMDB0002000	0.87 (0.28, 1,58)	0.004	0.219
Deoxycholate isomer-C	Unavailable	0.72 (0.11, 1.32)	0.021	0.388
C18:0 LPC plasmalogen	HMDB0011149	0.72 (0.14, 1.29)	0.015	0.388
C18:1 LPC plasmalogen	HMDB0011149	0.70 (0.13, 1.26)	0.015	0.388
C18:2 SM	HMDB0012101	1.06 (0.51, 1.61)	1.76 E-04	**0.052**
diHOME-B	Unavailable	0.66 (0.07, 1.25)	0.028	0.413
Glycoursodeoxycholate	HMDB0000708	0.66 (0.05, 1.28)	0.034	0.439
Trimethylamine-N-oxide	HMDB0000925	0.58 (0.03, 1.14)	0.038	0.439
Dimethylglycine	HMDB0000092	0.55 (0.02, 1.07)	0.040	0.439
Deoxycholate isomer-G	Unavailable	0.59 (0.01, 1.17)	0.046	0.466

[1]All values are beta estimates obtained from multivariable-adjusted linear regression modeling 5-unit increments of EDIH as the main predictor of interest and metabolite as the main response variable of interest.
[2]Models were adjusted for body mass index (continuous) age, physical activity, educational level, race/ethnicity, aspirin/NSAIDs use, smoking status, WHI Hormone Therapy trial arm, CHD case-control status.
[3]Statistical significance was defined as false-discovery rate adjusted $p<0.10$.

Table S3. Metabolites associated with EDIH at an FDR-adjusted P<0.20 in the discovery and replication datasets, among overweight or obese women[1,2,3]

		**Associations in WHI-HT (discovery, *n*=792)**		**Associations in WHI-OS (replication, *n*=497)**	
**Metabolite**	**HMDB ID**	**Beta estimate (95%CI)**	**FDR-adjusted P-value**	**Beta estimate (95%CI)**	**FDR-adjusted P-value**
**Trigonelline**	HMDB0000875	-0.62 (-0.94, -0.29)	0.032	-0.75 (-1.20, -0.31)	**0.013**
**C14:0 CE**	HMDB0006725	-0.44 (-0.79, -0.09)	0.109	-0.66 (-1.09, -0.24)	**0.013**
**C16:1 CE**	HMDB0000658	-0.47 (-0.79, -0.14)	0.095	-0.81 (-1.25, -0.36)	**0.012**
**C18:1 CE**	HMDB0000918	-0.47 (-0.81, -0.13)	0.103	-0.55 (-1.02, -0.09)	**0.032**
C18:2 CE	HMDB0000610	-0.33 (-0.66, -0.01)	0.174	-0.19 (-0.67, 0.29)	0.437
**C18:3 CE**	HMDB0010370	-0.43 (-0.77, -0.09)	0.109	-0.52 (-0.98, -0.07)	**0.037**
C20:1 CE	HMDB0005193	-0.46 (-0.80, -0.11)	0.109	0.03 (-0.40, 0.45)	0.906
C20:2 CE	HMDB0006734	-0.38 (-0.71, -0.06)	0.134	-0.16 (-0.61, 0.29)	0.481
**C20:3 CE**	HMDB0006736	-0.52 (-0.86, -0.19)	0.061	-0.43 (-0.90, 0.04)	**0.072**
C20:4 CE	HMDB0006726	-0.42 (-0.76, -0.09)	0.109	-0.15 (-0.63, 0.32)	0.526
**C20:5 CE**	HMDB0006731	-0.42 (-0.75, -0.10)	0.109	-0.31 (-0.77, 0.16)	0.194
C22:4 CE	HMDB0006729	-0.33 (-0.68, 0.01)	0.193	-0.07 (-0.53, 0.41)	0.785
C22:6 CE	HMDB0006733	-0.35 (0.03, 0.69)	0.170	-0.06 (-0.51, 0.39)	0.797
X4_pyridoxate	Unavailable	-0.31 (-0.64, 0.01)	0.193	-0.68 (-1.10, -0.26)	**0.013**
C14:0 LPC-A	HMDB0010379	-0.38 (-0.73, -0.02)	0.170	-0.54 (-0.99, -0.10)	**0.032**
C16:0 LPC	HMDB0010382	-0.36 (-0.72, -0.001)	0.184	-0.32 (-0.78, 0.14)	0.168
C16:1 LPC	HMDB0010383	-0.48 (-0.83, -0.13)	0.103	-0.64 (-1.08, -0.20)	**0.018**
C16:1 LPC plasmalogen	Unavailable	-0.37 (-0.73, -0.02)	0.171	-0.06 (-0.51, 0.38)	0.781
C18:1 LPC	HMDB0002815	-0.43 (-0.78, -0.09)	0.109	-0.28 (-0.73, 0.17)	0.220
C20:1 LPC	HMDB0010391	-0.64 (-1.01, -0.27)	0.032	-0.29 (-0.68, -0.11)	0.156
C20:2 LPC	HMDB0010392	-0.40 (0.82, 0.009)	0.193	-0.27 (-0.62, 0.07)	0.124
C20:3 LPC	HMDB0010393	-0.40 (-0.76, -0.04)	0.150	-0.31 (-0.75, 0.12)	0.155
C20:4 LPC	HMDB0010395	-0.38 (-0.74, -0.02)	0.171	-0.07 (-0.50, 0.37)	0.767
C22:6 LPC	HMDB0010404	-0.37 (-0.72, -0.02)	0.172	-0.11 (-0.53, 0.30)	0.602

C24:0 LPC	HMDB0008038	-0.39 (-0.72, -0.05)	0.140	-0.59 (-1.02, -0.16)	**0.020**
**C36:1 PS plasmalogen**	Unavailable	-0.45 (-0.82, -0.09)	0.109	-0.56 (-0.99, -0.13)	**0.025**
C16:0 LPE	HMDB0011503	-0.37 (-0.72, -0.02)	0.170	-0.55 (-0.98, -0.11)	**0.032**
C20:1 LPE	HMDB0011512	-0.43 (-0.84, -0.02)	0.172	-0.09 (-0.49, 0.30)	0.637
C22:6 LPE-B	HMDB0011526	-0.34 (-0.70, 0.01)	0.193	-0.26 (-0.67, 0.14)	0.202
**Eicosapentaenoate**	HMDB0001999	-0.53 (-0.84, -0.22)	0.032	-0.45 (-0.91, 0.01)	**0.067**
C32:1 PC plasmalogen-A	HMDB0013404	-0.34 (-0.70, 0.01)	0.193	-0.37 (-0.77, 0.02)	**0.071**
C34:2 PC plasmalogen-B	HMDB0011210	-0.39 (-0.72, -0.06)	0.134	-0.36 (-0.77, 0.05)	**0.087**
C40:10 PC	HMDB0008511	-0.35 (-0.70, 0.005)	0.191	-0.21 (-0.61, 0.18)	0.283
Proline betaine	HMDB0004827	-0.41 (-0.75, -0.08)	0.109	-0.33 (-0.77, 0.12)	0.146
Pipecolic acid	HMDB0000716	-0.33 (-0.66, 0.004)	0.191	-0.34 (-0.85, -0.16)	0.179
N-methylproline	HMDB0094696	-0.50 (-0.83, -0.18)	0.061	-0.31 (-0.78, 0.15)	0.183
Cortisol	HMDB0000063	-0.40 (-0.75, -0.06)	0.134	-0.31 (-0.76, 0.15)	0.183
C14:0 SM	HMDB0012097	-0.45 (-0.79, -0.10)	0.109	-0.41 (-0.85, 0.03)	**0.071**
C24:1 SM	HMDB0012107	-0.44 (-0.78, -0.11)	0.109	-0.31 (-0.79, 0.16)	0.197
Serotonin	HMDB0000259	-0.34 (-0.68, -0.01)	0.174	-0.23 (-0.77, 0.32)	0.409
Glycodeoxycholate	HMDB0000631	0.36 (0.03, 0.69)	0.170	-0.41 (-0.84, 0.03)	**0.071**
C23:0 Ceramide (d18:1)	HMDB0000950	0.37 (0.01, 0.73)	0.172	0.45 (0.03, 0.88)	**0.045**
2-aminooctanoate	HMDB0000991	0.35 (0.01, 0.69)	0.172	0.25 (-0.26, 0.77)	0.334
C22:0 Ceramide (d18:1)	HMDB0004952	0.36 (0.005, 0.72)	0.179	0.20 (-0.22, 0.63)	0.349
Butyrobetaine	HMDB0006831	0.34 (0.01, 0.66)	0.174	0.21 (-0.29, 0.72)	0.405
1-methyladenosine	HMDB0003331	0.39 (0.07, 0.71)	0.122	0.20 (-0.29, 0.70)	0.413
2-hydroxyhexadecanoate	HMDB0031057	0.43 (0.09, 0.77)	0.109	0.17 (-0.27, 0.61)	0.447
Cystathionine	HMDB0000099	0.55 (0.23, 0.86)	0.032	-0.14 (-0.56, 0.28)	0.501
cAMP	HMDB0000058	0.49 (0.20, 0.78)	0.032	-0.08 (-0.68, 0.51)	0.784
Oleate	HMDB0000207	0.34 (-0.001, 0.68)	0.184	0.38 (-0.09, 0.85)	0.110
C4-OH carnitine	HMDB0013127	0.48 (0.17, 0.79)	0.061	0.40 (-0.04, 0.83)	**0.076**
C6 carnitine	HMDB0000705	0.40 (0.07, 0.72)	0.117	0.77 (0.29, 1.25)	**0.013**
C7 carnitine	HMDB0013238	0.35 (0.07, 0.63)	0.109	0.77 (0.27, 1.27)	**0.013**
C9 carnitine	HMDB0013288	0.36 (0.02, 0.70)	0.170	0.55 (0.10, 1.01)	**0.032**
**C10:2 carnitine**	HMDB0013325	0.63 (0.28, 0.97)	0.032	0.60 (0.17, 1.04)	**0.020**
C12:1 carnitine	HMDB0013326	0.33 (-0.009, 0.66)	0.193	0.43 (-0.03, 0.89)	**0.071**

C14:2 carnitine	HMDB0013331	0.37 (0.04, 0.70)	0.149	0.52 (0.05, 0.98)	**0.039**
C36:1 DAG	HMDB0007216	0.38 (0.05, 0.71)	0.143	0.26 (-0.18, 0.70)	0.250
**C36:3 DAG**	HMDB0007219	0.43 (0.09, 0.77)	0.109	0.55 (0.09, 1.01)	**0.032**
**C36:4 DAG-A**	HMDB0007248	0.52 (0.17, 0.86)	0.062	0.61 (0.17, 1.05)	**0.020**
C50:1 TAG	HMDB0044109	0.33 (0.02, 0.65)	0.171	0.12 (-0.34, 0.59)	0.611
C50:4 TAG	HMDB0005435	0.37 (0.05, 0.70)	0.143	0.06 (-0.41, 0.54)	0.795
C51:0 TAG	HMDB0031106	0.33 (-0.02, 0.67)	0.196	0.12 (-0.31, 0.54)	0.592
**C51:3 TAG**	Unavailable	0.47 (0.14, 0.81)	0.095	0.63 (0.20, 1.07)	**0.018**
C52:0 TAG	HMDB0005365	0.39 (0.02, 0.76)	0.171	-0.01 (-0.40, 0.37)	0.953
C52:1 TAG	HMDB0005367	0.41 (0.09, 0.73)	0.109	0.18 (-0.28, 0.63)	0.448
C52:2 TAG	HMDB0005369	0.36 (0.04, 0.69)	0.149	0.33 (-0.11, 0.78)	0.144
**C52:3 TAG**	HMDB0005384	0.46 (0.11, 0.80)	0.109	0.49 (0.05, 0.92)	**0.039**
**C52:4 TAG**	HMDB0005363	0.61 (0.26, 0.95)	0.032	0.56 (0.11, 1.00)	**0.032**
C54:1 TAG	HMDB0005395	0.41 (0.07, 0.74)	0.130	0.12 (-0.32, 0.57)	0.581
C54:2 TAG	HMDB0005403	0.41 (0.08, 0.75)	0.109	0.35 (-0.09, 0.80)	0.120
**C54:3 TAG**	HMDB0005405	0.36 (0.02, 0.70)	0.171	0.43 (-0.02, 0.88)	**0.071**
**C54:4 TAG**	HMDB0005370	0.40 (0.06, 0.75)	0.134	0.53 (-0.07, 0.99)	**0.037**
**C54:6 TAG**	HMDB0005391	0.49 (0.14, 0.84)	0.095	0.34 (-0.10, 0.79)	0.133
C56:5 TAG	HMDB0005406	0.37 (00, 0.74)	0.184	-0.36 (-0.81, 0.09)	0.114
C56:8 TAG	HMDB0005392	0.45 (0.11, 0.79)	0.109	-0.38 (-0.81, 0.06)	**0.090**
Pseudouridine	HMDB0000767	0.33 (-0.02, 0.67)	0.196	-0.11 (-0.53, 0.31)	0.595
Piperine	HMDB0029377	0.32 (-0.02, 0.65)	0.196	0.11 (-0.32, 0.54)	0.619
N4-acetylcytidine	HMDB0005923	0.54 (0.21, 0.86)	0.037	0.05 (-0.39, -0.48)	0.826
Phenylacetylglutamine	HMDB0006344	0.30 (0.03, 0.57)	0.149	0.06 (-0.54, 0.66)	0.844
Isoleucine	HMDB0000172	0.57 (0.26, 0.88)	0.032	0.31 (-0.13, 0.75)	0.162
Valine	HMDB0000883	0.28 (0.04, 0.52)	0.134	0.15 (-0.41, 0.72)	0.595
Proline	HMDB0000162	0.38 (0.05, 0.71)	0.141	0.0002 (-0.47, 0.47)	0.999

[1]All values are beta estimates obtained from multivariable-adjusted linear regression modeling 5-unit increments of EDIH as the main predictor of interest and metabolite as the main response variable of interest.
[2]Models were adjusted for body mass index (continuous) age, physical activity, educational level, race/ethnicity, aspirin/NSAIDs use, smoking status, WHI Hormone Therapy trial arm, CHD case-control status.
[3]Statistical significance was defined as false-discovery rate adjusted $p<0.10$.
